# Disruption of adaptive immunity does not attenuate disease in the *Ndufs4*(-/-) model of Leigh syndrome

**DOI:** 10.1371/journal.pone.0324268

**Published:** 2025-06-10

**Authors:** Allison R. Hanaford, Asheema Khanna, Vivian Truong, Katerina James, Ryan Liao, Yihan Chen, Michael Mulholland, Ernst-Bernhard Kayser, Kino Watanabe, Erin Shien Hsieh, Philip G. Morgan, Surojit Sarkar, Vandana Kalia, Simon C. Johnson

**Affiliations:** 1 Center for Integrative Brain Research, Seattle Children’s Research Institute, Seattle, Washington, United States of America; 2 Ben Towne Center for Childhood Cancer and Blood Disorders Research, Seattle Children’s Research Institute, Seattle, Washington, United States of America; 3 Department of Applied Sciences, Translational Bioscience, Northumbria University, Newcastle, United Kingdom; 4 Department of Anesthesiology and Pain Medicine, University of Washington, Seattle, Washington, United States of America; 5 Department of Paediatrics, University of Washington School of Medicine, Seattle, Washington, United States of America; Imagine Institute, FRANCE

## Abstract

Leigh syndrome (LS) is the most common pediatric presentation of genetic mitochondrial disease and characterized by neurological and metabolic abnormalities. The hallmark of the disease is the presence of progressive, bilateral, symmetric neurodegenerative lesions in the brainstem and/or basal ganglia. Recent studies in the *Ndufs4*(-/-) mouse model of LS indicate that disease is causally driven by the immune system. Both microglia and peripherally originating macrophages are enriched in the lesions of *Ndufs4*(-/-) mice and pharmacologic elimination of these cell types prevents disease indicating a crucial role for innate immune cells. Here, we investigated the role of the adaptive immune system in *Ndufs4*(-/-) disease pathogenesis. We crossed *Ndufs4*(-/-) mice with mice expressing a null form of interleukin 2 receptor gamma (*Il2rg*) and monitored disease onset and progression. *Il2rg* knockout (KO) mice have dramatically depleted numbers of B-, T-, the adaptive immune system’s key cellular actors, and NK-cells. We observed no difference in neurological disease progression or overall survival between *Ndufs4*(-/-)/*Il2rg*(WT) and *Ndufs4*(-/-)/*Il2rg*(KO) mice, strongly suggesting that T cells, B cells, and NK cells do not play a significant role in CNS disease pathogenesis in *Ndufs4*(-/-) mice. Combined with previous studies indicating a causal role for macrophages, we conclude that LS CNS pathology is primarily driven by the monocyte/macrophage innate immune system.

## Introduction

Leigh syndrome (LS) is the most common clinical pediatric presentation of genetic mitochondrial disease (GMD). Also known as subacute necrotizing encephalomyopathy, the condition is characterized by symmetric, progressive, bilaterial necrotic lesions in the brainstem, basal ganglia, and occasionally cerebellum [1,2]. Brainstem lesions lead to respiratory failure — the most common cause of death for patients [3]. Symptoms typically begin in early childhood, often after a fever or viral infection [3–6]. Symptoms can include seizures, lactic acidosis, ataxia, and hypotonia.

LS is genetically diverse. Mutations in over 110 genes, in both the nuclear and mitochondrial genomes, have been causally linked to LS [7,8]. Mutations in *NDUFS4*, which encodes an electron transport chain complex I structural/assembly protein, are one cause of LS in humans [9]. Mice homozygous for *Ndufs4* loss of function alleles develop a disease highly consistent with that observed in humans. These animals are born healthy but begin to show signs of progressive neurodegeneration early in life and have a significantly shortened life span (median survival is around 60 days) [10]. *Ndufs4* null (termed knockout (KO), or (-/-)) mice develop bilateral, necrotic, inflammatory lesions in the brainstem, cerebellum and olfactory bulb, similar to those seen in humans [10].

Using the *Ndufs4*(-/-) mouse model, we have previously shown that these lesions are enriched with macrophages of peripheral origin in addition to microglia, brain resident macrophages [11]. Notably, while the exclusive genetic elimination of microglia only modestly improves the survival of *Ndufs4*(-/-) mice, combined pharmacologic depletion of both microglia and circulating macrophages with the Csf1r inhibitor pexidartinib fully suppresses neurodegenerative lesions, prevents neurodegenerative symptoms, and dramatically prolongs lifespan [11,12]. Pexidartinib treated animals do not present with LS-like symptoms before death, ultimately dying of apparent drug toxicity from chronic pexidartinib treatment [12]. Additionally, we recently showed that loss of interferon gamma (IFNγ) modestly improves survival and delays disease in *Ndufs4*(-/-) mice [13].

While these findings demonstrate that disease in the *Ndufs4*(-/-) mouse is directly mediated by immune cells, and most strongly implicate macrophages and monocytes (see ***Discussion***), the role of other immune cells in disease pathogenesis remains unclear. In particular, the role, if any, of adaptive immune T-, B-, and NK cells has not yet been directly assessed. Abnormalities in humoral and T-cell responses have been identified in multiple patients with GMDs [4]. A case series of Leigh syndrome patients found that all had immunological deficiencies—including deficient response to vaccination, reduced class switched B-cells, and reduced memory T-cells [14].

*IL2RG* encodes the IL-2R gamma chain, a subunit common to multiple interleukin receptors (such as IL-2, IL-4, IL-7, IL-15, IL-9, IL-21). IL-2Rg is a critical component of receptors (such as IL-2, IL-15, IL-7) responsible for differentiation and survival of T-, B-, and NK- cells [15]. In humans, *IL2RG* defects can cause X-linked severe combined immunodeficiency [16]. Mice lacking functional *Il2r*g have significantly reduced levels of B, T, and NK-cells [17]. Il2rg loss of function can be combined with loss of function in other immune genes (such as *Prkdc* or *Rag2*) to generate highly immune deficient mouse strains for xenografting and generation of “humanized” mice [18]. Here, we generated *Ndufs4*(-/-)/*Il2rg*(KO) double knockout mice in order to directly assess whether NK cells and adaptive immune cells contribute to disease pathogenesis in LS.

## Materials and methods

### Animals

*Il2rg* knockout (KO) mice are from the Jackson Lab (strain 003174). *Il2rg* is on the X chromosome, so *Il2rg*(KO) indicates a homozygous mutant female (*IL2rg*(X+/X-)) or hemizygous mutant male (*IL2rg*(X-/Y)). *Ndufs4*(+/-) mice were originally obtained from the Palmiter laboratory at University of Washington, Seattle, Washington USA, and are available from the Jackson Laboratory (strain 027058). Strain details are described in Kruse et al [10]. Both lines are on the C57BL/6 background. *Ndufs4*(-/-) mice cannot be used for breeding due to their short lifespan and severe disease. *Ndufs4*(+/-) mice were bred with *Il2rg*(KO) mice to produce *Ndufs4*(+/-)/*IL2rg*(X+/X-) females and *Ndufs4*(+/-)*/Il2rg*(X-/Y) males which were then crossed to produce *Ndufs4*(-/-)/*Il2rg*(X-/X-) females, *Ndufs4*(-/-)/*Il2rg(*X*+/*X-) females, and *Ndufs4*(-/-)/*Il2rg*(X-/Y) males. Genotyping of the *Ndufs4* and *Il2rg* alleles were performed according to the Jackson laboratory methods (strains 003174 and 027058). Only mice with PCR confirmed genotype were included in the study.

Mice were weaned at P20-22 days of age. *Ndufs4*(-/-) animals were housed with control littermates for warmth as *Ndufs4*(-/-) mice have low body temperature [10]. Mice were weighed and health assessed a minimum of 3 times a week. Following onset of *Ndufs4*(-/-) symptoms, wet food was provided in the bottom of the cage. Animals were euthanized if they lost 20% of maximum body weight for two consecutive days, were immobile, or were found moribund [12,19]. These endpoints have been developed to minimize suffering. Mice heterozygous for *Ndufs4* loss of function have no reported phenotype, so controls consisted of both heterozygous and wild-type (WT) *Ndufs4* animals. We refer to *Ndfus4*(Ctrl) mice here for clarity. The *Ndufs4*(Ctrl) and *Ndufs4*(-/-) mice wild type for *Il2rg* used in this study came from crosses of *Ndufs4*(+/-)/*Il2rg*(-/+) female mice with *Ndufs4*(+/-)/*Il2rg*(+/Y) male mice and our general *Ndufs4* colony. Mice were fed PicoLab Diet 5058 and were on a 12-hour light-dark cycle. All animal experiments followed Seattle Children’s Research Institute (SCRI) guidelines and were approved by the SCRI IACUC. Euthanasia was performed by approved methods – cervical dislocation or CO_2_ asphyxiation.

Clasping and ataxia were assessed by visual scoring and analyzed as previously described [19]. During disease progression, *Ndufs4*(-/-) animals can display intermittent/transient improvement of symptoms, so here we report whether the animal *ever* displayed the symptoms for two or more consecutive days. Onset of weight loss is reported as the age of maximum body weight.

A Med Associates ENV-571M single-lane rotarod was used for the rotarod performance test. A mouse was placed on the rod already rotating at 6 rpm and latency to fall was timed for a maximum of 600 seconds while rotation remained constant. For each mouse, three trials were performed with a minimum of 5 minutes between each trial. The best of three trials was reported.

### Lymphocyte analysis

Blood from adult *Ndufs4*(+/+) mice between ages P39 and P83 was collected by terminal cardiac puncture and placed in 0.4% sodium citrate to prevent coagulation. Peripheral blood mononuclear cells (PBMCs) were isolated using lymphocyte separation medium from Corning. Isolated PBMCs were stained for surface antigens (CD45, NK1.1, B220, and CD3) using fluorescent antibodies and dead cells were stained with Zombie Live/Dead dye from BioLegend (cat. #423101, used at a 1:200 dilution). Antibody clones, fluorochromes, and dilutions for staining are provided in Table 1. Staining was performed on ice for 45 minutes in the dark. Stained cells were acquired using a LSRII Fortessa Cell Analyzer (BD Biosciences). Data was analyzed using FlowJo V9.0 software.

**Table 1:** Antibody clones, fluorochromes, and dilutions.

Name	Fluor	Clone	Dilution
anti-CD45	AF488	30-F11	1:100
anti-CD3	APC	145-2C11	1:100
anti-B220	PE	RA3-6B2	1:100
anti-NK1.1	PE-Cy7	PK136	1:100

### Statistical analysis

**Fig 1:**
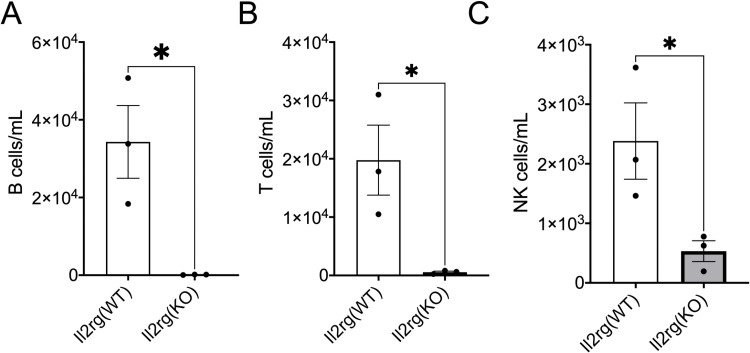
Mice expressing a null form of *Il2rg* lack lymphocytes. Peripheral blood mononuclear cells (PBMCs) were isolated from the blood of adult mice of the indicated genotype, stained with fluorescent antibodies, and analyzed by flow cytometry. Within the live cell population, leukocytes were identified by positive staining for CD45, the common leukocyte antigen. Among CD45 positive cells, T-, B-, and NK-cells were identified by staining for CD3, B220 or NK1.1, respectively. B- (A), T- (B), and NK- cells (C) were significantly depleted in *ll2rg*(KO) mice compared to *Il2rg*(WT) mice (data shown are cells per 1mL of blood; for gating and normalization to CD45 positive cell counts see S1 Fig). (A-C) *p < 0.05 by unpaired parametric t-test (see Methods). n = 3 animals per genotype, each datapoint represents an individual mouse. Bars show mean and error bars standard error of the mean (SEM). See S1–S3 Files for raw data.

**Fig 2:**
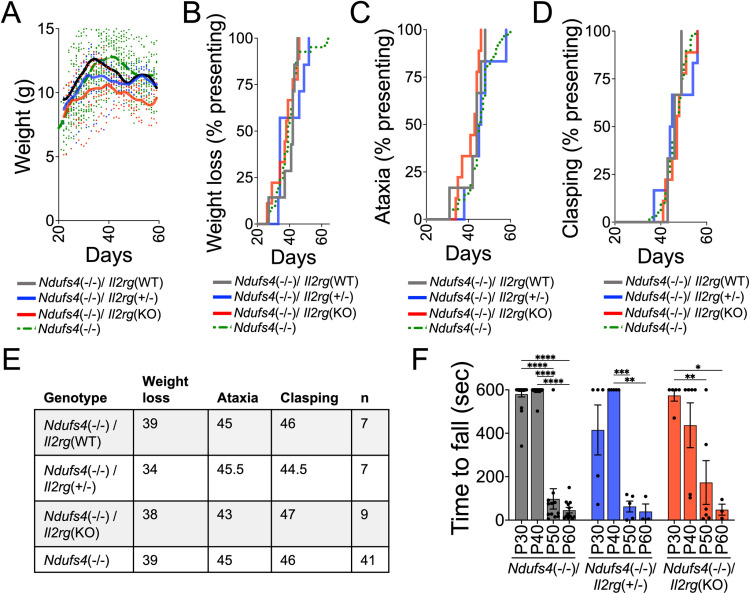
Loss of *Il2rg* does not impact disease course in *Ndufs4*(-/-) mice. (A) Weight of *Ndufs4*(-/-)/*Il2rg*(WT), *Ndufs4*(-/-)/*Il2rg*(+/-), and *Ndufs4*(-/-)/*Il2rg*(KO) animals. Weight from our complete *Ndufs4*(-/-) colony—*Ndufs4(-/-)*/*Il2rg*(WT) mice from our *Ndufs4*(-/-)/*Il2rg*(+/-) colony and those never crossed with *Il2rg*(KO)--provided for reference. Data shown Locally Weighted Scatterplot Smoothing (LOWESS) curves with all datapoints (descriptive only, not statistical comparisons). (B) Age of onset of weight loss (see Methods). Pairwise log-rank comparisons = no significant findings between any pairwise comparison. (C) Onset of ataxia onset. *Ndufs4*(-/-)/*Il2rg*(+/-) vs *Ndufs4*(-/-)/*Il2rg*(KO) *p < 0.05 by Log-rank test, other comparisons not significant. (D) Onset of forelimb clasping. Pairwise log-rank comparisons = no significant findings between any pairwise comparison. (E) Summary of median age of symptom onset. (F) Rotarod performance as assessed by latency to fall (see Methods). Comparisons were made by two-way ANOVA followed by all possible pairwise t-tests with Tukey’s multiple testing correction. No significant differences between genotypes at any age. *p < 0.05, **p < 0.005, ***p < 0.0005, ****p < 0.0001. Note that comparisons have been made to the larger *Ndufs4*(-/-) cohort which includes *Ndufs4*(-/-)/*Il2rg*(WT) mice and those never crossed with *Il2rg*(KO). (A-E) No significant differences were observed between *Ndufs4*(-/-)/*Il2rg*(WT) (mice birthed from *Ndufs4*(-/-)/*Il2rg*(+/-) colony)) and our complete *Ndufs4*(-/-) colony--*Ndufs4*(-/-)/*Il2rg*(WT) mice and those never crossed with *Il2rg*(KO).

**Fig 3:**
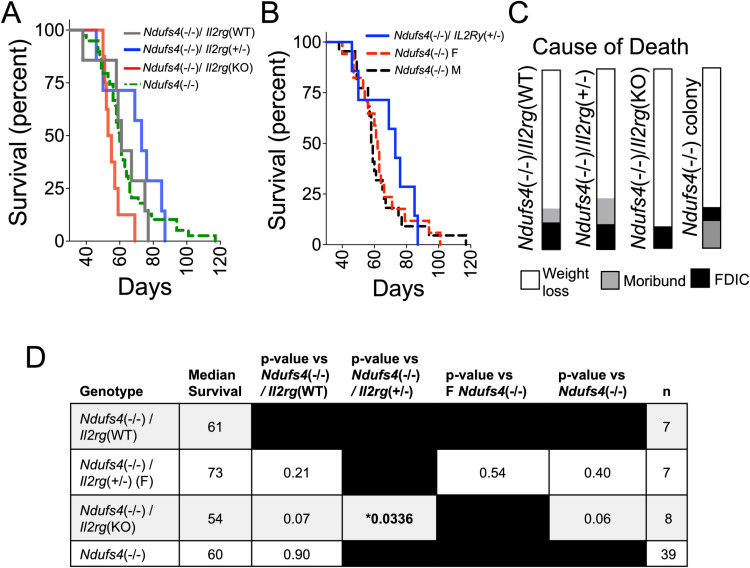
*Il2rg does not impact survival of* Ndufs4*(-/-) mice.* (A) Survival curves for *Ndufs4*(-/-)/*Il2rg*(WT), *Ndufs4*(-/-)/*Il2rg*(+/-), and *Ndufs4*(-/-)/*Il24g*(KO) animals, with our larger *Ndufs4*(-/-) cohort - *Ndufs4*(-/-)/*Il2rg*(WT) mice and those never crossed with *Il2rg*(KO) – provided for reference. See (D) for statistical information (p-values by pairwise log-rank comparisons). (B) Data from (A), plotting *Ndufs4*(-/-)/*Il24g*(+/-) (all female as *Il2rg* is X-linked, see description of genotype in Methods) plotted against sex-split curves for *Ndufs4*(-/-) mice. Statistical comparison between female groups provided in (D), no significant differences observed. (C) The primary cause of death in *Ndufs4*(-/-)/*Il2rg*(WT), *Ndufs4*(-/-)/*Il2rg*(+/-), *Ndufs4*(-/-)/*Il2rg*(KO), and the larger *Ndufs4*(-/-) cohort was euthanasia due to weight loss, with similar patterns observed in all groups (n’s in (D)). FDIC – found dead in cage (cause of death not known). (D) Summary of median survival, number of animals per group, and statistical comparisons by Log-rank test. Only *Ndufs4*(-/-)/*Il24g*(KO) vs *Ndufs4*(-/-)/*Il2rg*(+/-) reached statistical significance by Log-rank test, *p = 0.034. *Ndufs4*(-/-)/*Il2rg*(WT) and *Ndufs4*(-/-) survival were not significantly different.

All statistical analyses were performed using GraphPad Prism 10.0.0 with statistical test results and replicate numbers detailed in figure legends. Error bars represent the standard error of the mean (SEM). Analyses included pairwise unpaired parametric t-test (Figs 1A–1C and S1B–S1D); two-way ANOVA followed by all possible pairwise t-tests with Tukey’s multiple testing correction (Fig 2F); and pairwise log-rank tests (Figs 2B–2D, 3A and 3B).

### Scientific rigor

*Sex-*Both male and female animals were used in these experiments. No significant sex differences have been reported in *Ndufs4*(KO)s or *Il2rg*(KO)s and none were observed.

### Exclusion criteria

Only animals with a PCR confirmed genotype were included in the study. Animals euthanized prior to the age of disease onset in the *Ndufs4*(-/-) were excluded from study. Our criteria for early life euthanasia and study exclusion include severe weaning stress (significant weight loss or death prior to P30), runts (defined as ≤5 g body weight at weaning age), or health issues unrelated to the *Ndufs4*(-/-) phenotype (such as hydrocephalus). These criteria are applied to all genotypes as part of our standard animal care. Exclusion due to these criteria occurs in up to ~10% of *Ndufs4*(-/-) animals in our prior studies. From *Ndufs4*(+/-)/*Il2rg*(+/-) x *Ndufs4*(+/-)/*Il2rg*(-/Y) and *Ndufs4*(+/-)/*Il2rg*(+/-) x Ndufs4(+/-)/Il2rg(+/Y) matings, 1 of 10 *Ndufs4*(-/-)/*Il2rg*(KO) and 1 of 8 *Ndufs4*(-/-)/*Il2rg*(WT) were excluded, similar to our overall exclusion rate for *Ndufs4*(-/-) mice.

## Results

### *Il2rg* knockout mice have reduced lymphocyte numbers

We first quantified B-, T-, and NK cells in the blood of adult *Il2rg*(KO) and *Il2rg*(WT) mice by flow cytometry in order to validate the previously reported impact of *Il2rg* depletion. *Il2rg* is encoded on the X chromosome, so *Il2rg*(KO) indicates homozygous knockout female (*IL2rg*(X+/X-)) or hemizygous knockout male (*IL2rg*(X-/Y)). Consistent with prior reports, *Il2rg*(KO) mice had a dramatic reduction in B-, T-, and NK-cells as indicated by a reduction in the absolute number of B220 positive, CD3 positive, and NK1.1 positive cells, respectively (Fig 1A–1C). Flow cytometry gating and cell frequency within the CD45 positive leukocyte population is shown in S1 Fig (see S1–S3 Files for raw cytometry data).

### Reduction of lymphocytes does not impact neurological phenotype in Ndufs4(-/-) mice

To investigate the contribution of lymphocytes to *Ndufs4*(-/-) pathology, we crossed *Ndufs4*(+/-) mice with *Il2rg*(KO) mice to generate an *Ndufs4*(+/-)/*Il2rg*(+/-) line for production of double knockouts. As in humans, mouse *Il2rg* is an X-linked gene; here, we use *Il2rg*(KO) to indicate both female homozygous knockouts and hemizygous *Il2rg* knockout *Il2rg* males, and *Il2rg*(WT) for homozygous wildtype females and hemizygous wildtype males. All *Il2rg(*+/-) heterozygotes are female.

Onset of weight loss is one of the first signs of disease observable in the *Ndufs4*(-/-) mouse model. *Il2rg* disruption was associated with a modest reduction in weight, in both *Ndufs4*(-/-) (Fig 2A). *Ndufs4(*Ctrl*)/Il2rg(KO)* mice are also smaller in size than *Ndufs4*(Ctrl*)/Il2rg(*WT*)* mice (S2 Fig). There was no difference in age of weight loss onset between the three genotypes, *Ndufs4(-/-)/Il2rg(KO)*, *Ndufs4(-/-)/Il2rg(+/-)*, and *Ndufs4*(-/-)/*Il2rg*(WT), and each displayed similar weight gain/loss patterns (Fig 2A and 2B). Weight from our Ndufs*4*(-/-) colony--*Ndufs4*(-/-)/*Il2rg*(WT) mice and those *Ndufs4*(-/-) mice never crossed with *Il2rg*(KO))-- is provided for reference

Ataxia and limb clasping are signs of neurological dysfunction assessed by visual scoring of symptoms in the *Ndufs4*(-/-) line. *Il2rg* deficiency had no impact on the age of onset of these symptoms (Fig 2C and 2D). The difference between age of onset of ataxia in *Ndufs4*(-/-)/*Il2rg(KO)* mice and *Ndufs4*(-/-)/*l2rg(WT)* mice was statistically significant, but so small it is unlikely to be biologically meaningful. The median age of onset of weight loss, ataxia, and clasping for each genotype is summarized in Fig 2E including the sample size (n). Data from our *Ndufs4*(-/-) colony--*Ndufs4*(-/-)/*Il2rg*(WT) mice and those Ndufs4(-/-) mice never crossed with *Il2rg*(KO))-- is provided for reference.

Finally, *Ndufs4*(-/-) mice experience a progressive decline in rotarod performance, a behavioral test measuring overall coordination and muscle strength, as disease progresses. Decline in rotarod performance was not attenuated by *Il2rg* loss (Fig 2F).

### Reduction of lymphocytes very modestly impacts survival in *Ndufs4*(-/-) mice

Complete loss of *Il2rg* in *Ndufs4*(KO) mice did not impact overall survival (Fig 3A and 3B). *Ndufs4*(-/-)/*Il2rg*(+/-) animals had modestly, but statistically significant, increased survival compared to *Ndufs4*(-/-)/*Il2rg*(KO) mice. There is no statistically significant difference between *Ndufs4*(-/-)/*Il2rg*(+/-) mice compared with *Ndufs4*(-/-)/*Il2rg*(WT) mice or our larger *Ndufs4*(-/-) colony. *Il2rg*(+/-) mice are not known to phenotypically differ from *Il2rg*(WT) mice (*17*). Thus, it is unlikely that *Il2rg* heterozygosity provides a survival benefit.

Median survival, sample size, and statistical comparisons are shown in Fig 3D. Data from our Ndufs*4*(-/-) colony - *Ndufs4*(-/-)/*Il2rg*(WT) mice and those *Ndufs4*(-/-) mice never crossed with *Il2rg*(KO)) – are provided for reference.

The overall cause of death distribution, which can reveal subtle changes in disease progression, was similar among all groups (Fig 3C). The primary cause of death in *Ndufs4*(-/-) mice was euthanasia due to weight loss, regardless of *Il2rg* status. Survival data and statistics are summarized in Fig 3D.

## Discussion

Here, we used a genetic model to assess the role of lymphocytes in the onset and progression of CNS disease in the *Ndufs4*(-/-) model of LS. We found that severe depletion of NK cells, and B-, T-, representing the primary cellular actors of the adaptive immune system, did not impact overall disease onset or progression in the *Ndufs4*(-/-) mouse model of LS.

Our previous studies have demonstrated a causal role for immune cells in the pathogenesis of LS, with observations to date most strongly implicating circulating and tissue resident macrophages [11–13]. It has long been recognized that microglia (the brain resident macrophage) are highly abundant in LS CNS lesions; we have recently demonstrated that macrophages of peripheral origin are also highly enriched in the lesions observed in diseased *Ndufs4*(-/-) mice [11]. In addition, we have found that depletion of microglia alone only modestly impacts disease course in the *Ndufs4*(-/-) model [11], whereas pan-macrophage ablation via high dose pexidartinib prevents disease onset and dramatically extends survival [12]. Together with our findings here, these data strongly suggest that adaptive immune cells are not a significant component of CNS disease pathogenesis in *Ndufs4*(-/-) mice. This is consistent with the model that CNS pathology in LS is driven by actions of the macrophage/monocyte innate immune system*.*

In recent work, we found that loss of IFNγ provided a modest but statistically significant and gene dose-dependent improvement in survival and delay in symptom onset in *Ndufs4*(-/-) mice [13]. Notably, cytotoxic lymphocytes, including T- and NK- cells, and cytotoxic macrophages are the major producers of IFNγ [20,21]. Given that IFNγ depletion attenuates disease progression, but B-, T-, and NK- cell depletion fails to alter disease course, it seems likely that disease-promoting IFNγ is macrophage-derived, representing positive feedback.

In the cohorts tested here, we observed a modest but statistically significant reduction in *Ndufs4*(-/-)/*Il2rg*(KO) survival compared to *Ndufs4*(-/-)/*Il2rg*(+/-) animals. This might indicate that the *Ndufs4*(-/-)/*Il2rg*(KO) mice are more frail than *Ndufs4*(-/-)/*Il2rg*(+/-) animals, which would be consistent with the possibility that adaptive immune depletion not only fails to prevent disease but is actively harmful to the Leigh syndrome model. It is also possible that loss of *Il2rg* impacts the phenotype of macrophages, possibly slightly accelerating disease through increased activation. This may be worth further study.

Recent evidence supports the notion that activation of the innate immune system may occur in multiple forms of mitochondrial disease [4,22,23]. Various contributing pathways have been implicated, such as type I interferon signaling, but no clear single immune-activating mediator has yet been identified. Interestingly, while CNS lesions in LS appear to be an innate immune phenomenon, clinical immunodeficiencies appear to be common among GMD patients, including those with LS [24]. In one cohort of GMD patients, almost half were diagnosed with an immune deficiency (*25*). Evidence indicates that GMD patients have an impaired humoral response to infection, and failure to develop and/or maintain antibodies titers in response to vaccination is common in this patient population [25,26]. T-cell mediated immune alterations have also been reported in GMD patients, such a reduction in T-cell repertoire in MELAS patients [27,28]. A patient with GMD caused by mtDNA deletion had reduced T, B, and NK cell levels and no evidence of multimorbidity with other immune deficiency syndromes [29]. These defects in adaptive immunity might be related to dysregulation of the monocyte/macrophage population. Recurrent infections are common in GMD patients, particularly in the respiratory tract [24]. Infection has been associated with episodes of underlying disease progression and can be life threatening—sepsis and pneumonia are the two of the most common causes of death in pediatric GMD patients [3].

Integrating findings in human patients with our current and previous findings in the *Ndufs4*(-/-) mouse suggests that innate and adaptive immune actors are both differentially impacted in GMD, and play distinct roles in disease onset and progression. Our results support efforts to identify targeted therapies aimed at suppressing CNS disease driving macrophages/monocytes, while suggesting that patient benefits may depend on the ability to limit off-target suppression of already disrupted adaptive immune functions.

## Supporting information

S1 Fig Supplemental flow cytometry data. (A) Gating used to identify leukocyte population. Leukocytes are identified by CD45 expression. B-cells are characterized by high B220 expression and negative CD3 expression. T-cells are characterized by high CD3 expression and negative B220 expression. NK cells are identified by high NK1.1 expression and negative CD3 expression. (B) Frequency of B220 positive cells among the CD45 cell population (B-cells). (C) CD45/CD3 positive cells (T-cells) and D) CD45/NK1.1 positive cells (NK cells). While raw NK cell counts are significantly reduced in *Il2rg*(KO) mice (Fig 1C) their frequency within the overall leukocyte population is increased.
(TIF)

S2 Fig *Ndufs4*(Ctrl) mouse weights. Weight of male *Ndufs4* control mice knockout or WT for *Il2rg* (all from the *Ndufs4*(+/-)/*Il2rg* colony). *Ndufs4* controls are genotype *Ndufs4*(+/+) or *Ndufs4*(+/-) as previous work has shown no differences between the two (see Methods). *Il2rg* is an X-linked gene, so male heterozygotes do not exist. Data shown are average with SEM and Locally Weighted Scatterplot Smoothing (LOWESS) curves to show overall trends.
(TIF)

S1 File FCS files - first batch containing source data for flow cytometry. (ZIP)

S2 File FCS files - second batch containing source data for flow cytometry. (ZIP)

S3 File FCS files - third batch containing source data for flow cytometry. (ZIP)

S1 Data This file contains all raw data used for generation of the figures appearing in the manuscript. (XLSX)
