# Supplementary figures and images for: Disruption of adaptive immunity does not attenuate disease in the Ndufs4(-/-) model of Leigh syndrome
Source: PLoS One. 2025 Jun 10;20(6):e0324268. doi: 10.1371/journal.pone.0324268 (PMC12151442; doi:10.1371/journal.pone.0324268)

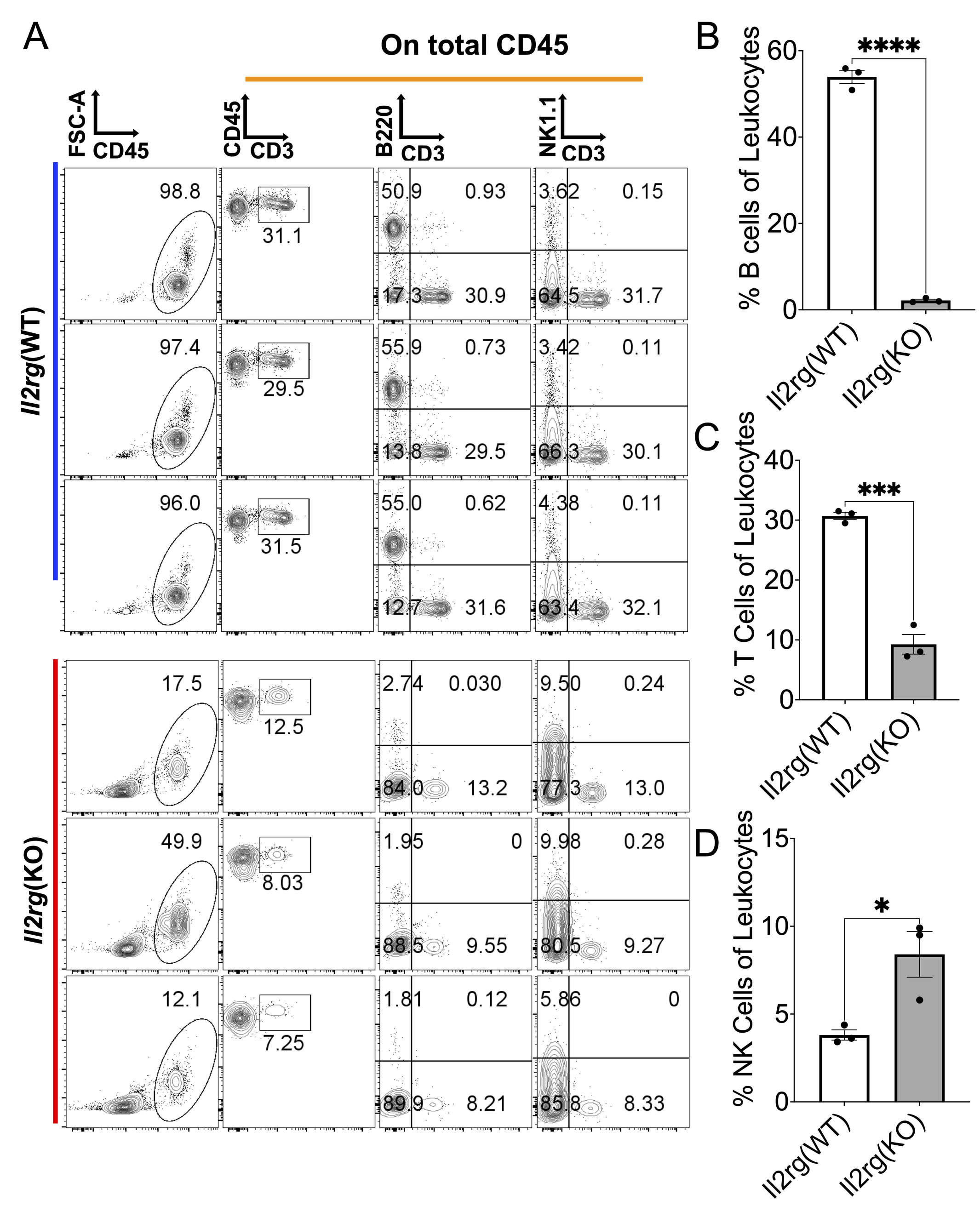

Supplement: S1 Fig — (A) Gating used to identify leukocyte population. Leukocytes are identified by CD45 expression. B-cells are characterized by high B220 expression and negative CD3 expression. T-cells are characterized by high CD3 expression and negative B220 expression. NK cells are identified by high NK1.1 expression and negative CD3 expression. (B) Frequency of B220 positive cells among the CD45 cell population (B-cells). (C) CD45/CD3 positive cells (T-cells) and D) CD45/NK1.1 positive cells (NK cells). While raw NK cell counts are significantly reduced in Il2rg(KO) mice (Fig 1C) their frequency within the overall leukocyte population is increased. (TIF) [file pone.0324268.s001.tif]

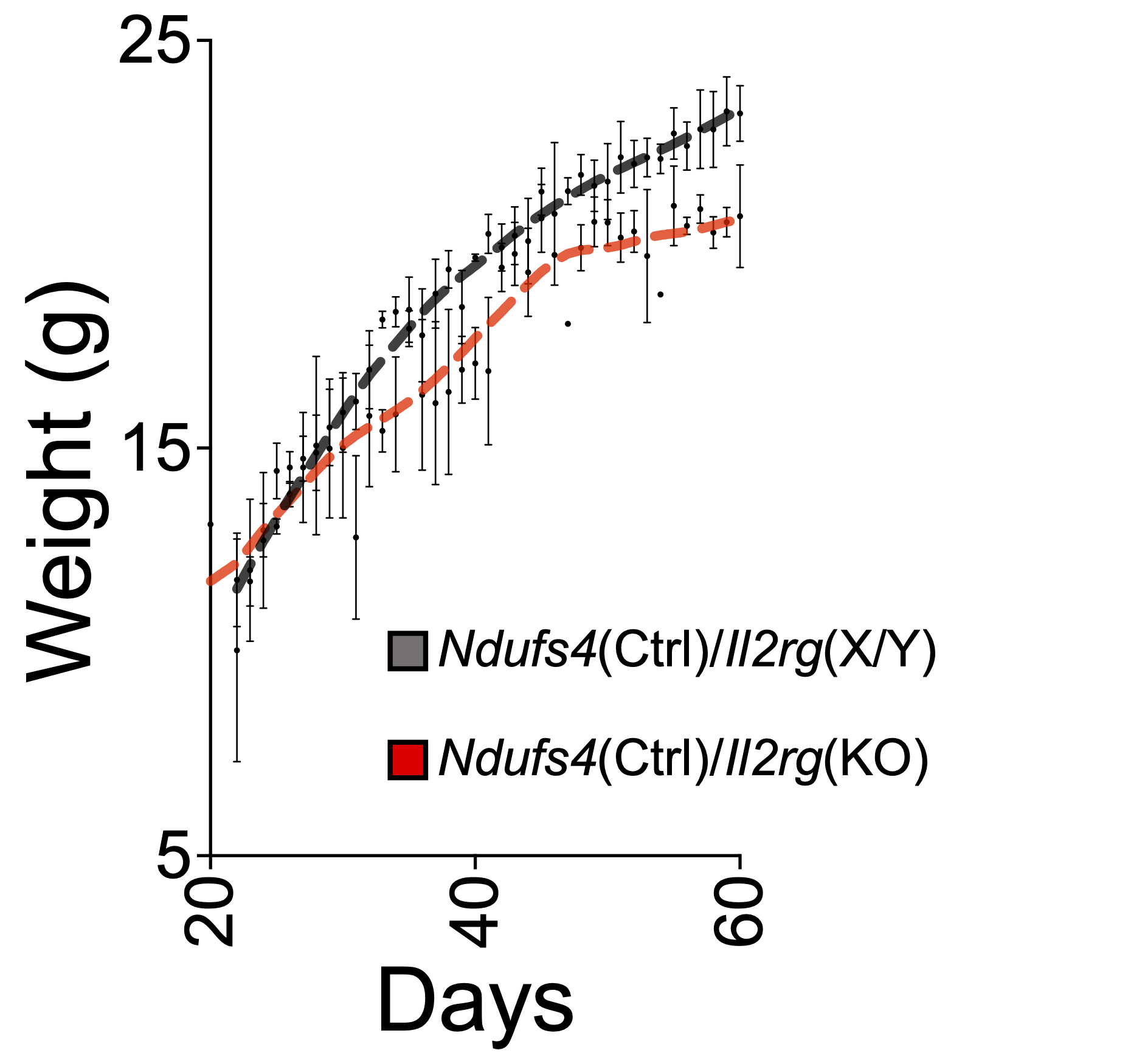

Supplement: S2 Fig — Weight of male Ndufs4 control mice knockout or WT for Il2rg (all from the Ndufs4(+/-)/Il2rg colony). Ndufs4 controls are genotype Ndufs4(+/+) or Ndufs4(+/-) as previous work has shown no differences between the two (see Methods). Il2rg is an X-linked gene, so male heterozygotes do not exist. Data shown are average with SEM and Locally Weighted Scatterplot Smoothing (LOWESS) curves to show overall trends. (TIF) [file pone.0324268.s002.tif]
