# Supplementary material for: Disruption of adaptive immunity does not attenuate disease in the Ndufs4(-/-) model of Leigh syndrome
Source: PLoS One. 2025 Jun 10;20(6):e0324268. doi: 10.1371/journal.pone.0324268 (PMC12151442; doi:10.1371/journal.pone.0324268)
Supplement: Attachment — Submitted filename: LS adaptive immunity.docx [file pone.0324268.s007.docx]

This short manuscript reported a negative finding that disruption of adaptive immunity by IL2Rg KO did not attenuate Leigh Syndrome mice with Ndufs4 KO. Reports of negative findings can be very important if there is a widespread and unproven belief. Unfortunately, I do not find a strong rationale for the proposed experiments. In other words, there is little evidence or clues that Leigh Syndrome will involve adaptive immunity. Adaptive immunity is very critical to protect against infection, spreading of cancer and its overactivity is associated with autoimmune diseases. Leigh Syndrome is a genetic disease involving mitochondria, presented as neurodegenerative, early in life. While a few manuscripts have been published to report the involvement of microglia (including the report by the current authors), the reasons to do such experiments are not strongly supported by previous literature. As such, the value of this short manuscript reporting negative findings is very limited. Most importantly, CNS has an immune privilege due to the blood brain barrier. It is unclear why they did a systemic KO of IL2Rg as there is no strong evidence of systemic inflammation in Leigh Syndrome. A more thoughtful experiments addressing mechanisms of neuroinflammation may be much more exciting.
