# Supplementary material for: Disruption of adaptive immunity does not attenuate disease in the Ndufs4(-/-) model of Leigh syndrome
Source: PLoS One. 2025 Jun 10;20(6):e0324268. doi: 10.1371/journal.pone.0324268 (PMC12151442; doi:10.1371/journal.pone.0324268)
Supplement: Attachment — Submitted filename: Responses to reviewer comments.docx [file pone.0324268.s010.docx]

Manuscript PONE-D-24-54394

Responses to reviewer comments

Overview:

We thank the reviewers for their thoughtful remarks. We have addressed the final comments from Reviewer 1 as follows, detailed point by point:

**Reviewer 1**

*Reviewer #1: The authors have satisfactorily addressed my main comments, which I thank them for.*

*I would just point out:*

*Although mention of Y is necessary, I am not sure mention of “X” in genotypes is necessary, now that the genotypes are well clarified.*

We thank the reviewer for their positive and thoughtful notes and careful review. Their feedback has been extremely productive throughout the review process.

As this point is optional, we have opted to keep X in the genotypes, as we feel it is helpful in keeping track of the genotypes and sexes.

*FACS antibodies should mention the clone and fluorochrome used.*

We apologize for this omission; we have added these details and the dilutions to the methods section as a supplemental table. They are as follows:

| **Name** | **Fluor** | **Clone** | **Dilution** |
| --- | --- | --- | --- |
| anti-CD45 | AF488 | 30-F11 | 1:100 |
| anti-CD3 | APC | 145-2C11 | 1:100 |
| anti-B220 | PE | RA3-6B2 | 1:100 |
| anti-NK1.1 | PE-Cy7 | PK136 | 1:100 |

We have also added the dilution and catalog number for the dead cell Zombie dye from BioLegend (cat. #423101, used at a 1:200 dilution).

*New version line 88: I think it should be “homozygous mutant female (IL2rg(X-/X-))” rather than (X+/X-)*

We thank the reviewer for catching this typo, it has been fixed.

*I also don’t understand this statement in the rebuttal, but I think KO should read +/- for females:
“In the remainder of the manuscript uses Ndufs4(+/-) and Il2rg(KO) to designate female hets (only females can be het, given the gene is X-linked) and KO (both male -/Y and -/- female) mice.”*

We apologize for the lack of clarity in our response statement; the relevant sentence in the text correctly states “Il2rg is encoded on the X chromosome, so Il2rg(KO) indicates homozygous knockout female (IL2rg(X+/X-)) or hemizygous knockout male (IL2rg(X-/Y)).” This was noted in the methods section and we have added it to the beginning of the results in this final revision. While there was a typo in our response, as the reviewer notes above the genotypes are well clarified in the last revision.

*New version line 154: I think the genotype of the male breeder should be Ndufs4(+/-) Il2rg (-/Y) and not Ndufs4(-/-) Il2rg (-/Y), as Ndufs4(-/-) animals can apparently not breed.*

We thank the reviewer for noting this typographical error; they are correct, and we have fixed the text.

*Line 207 should refer to Fig 3D I think.*

We thank the reviewer for noting this typographical error; they are correct, and we have fixed the text.

*New version line 242: the main word is missing (“survival”?)*

We thank the reviewer for noting this typographical error; they are correct, and we have fixed the text.

*I think figure 3B and Supp 3 are the same (and fig supp 3 not referred to by the text)*

This is correct, we appear to have accidentally left a duplicate of this panel in the manuscript file as supplemental, after it was included in the main figure. This duplicate has now been removed.
