# Supplementary material for: Disruption of adaptive immunity does not attenuate disease in the Ndufs4(-/-) model of Leigh syndrome
Source: PLoS One. 2025 Jun 10;20(6):e0324268. doi: 10.1371/journal.pone.0324268 (PMC12151442; doi:10.1371/journal.pone.0324268)
Supplement: Peer review history

# Author comment: Author response to Decision Letter 0

**DOI:** 10.1371/journal.pone.0324268.r001

26 Nov 2024

# Peer review: Decision Letter 0

**DOI:** 10.1371/journal.pone.0324268.r002

**Authors:** Metodi Metodiev

16 Jan 2025

Dear Dr. Johnson,

Thank you for submitting your manuscript to PLOS ONE. After careful consideration, we feel that it has merit but does not fully meet PLOS ONE’s publication criteria as it currently stands. Therefore, we invite you to submit a revised version of the manuscript that addresses the points raised during the review process.

The reviewers acknowledge the work’s significance, but require further changes in the form of minor revisions. Please address all points raised by the reviewers.

Please submit your revised manuscript by Mar 02 2025 11:59PM. If you will need more time than this to complete your revisions, please reply to this message or contact the journal office at plosone@plos.org . When you're ready to submit your revision, log on to https://www.editorialmanager.com/pone/ and select the 'Submissions Needing Revision' folder to locate your manuscript file.

- A rebuttal letter that responds to each point raised by the academic editor and reviewer(s). You should upload this letter as a separate file labeled 'Response to Reviewers'.
- A marked-up copy of your manuscript that highlights changes made to the original version. You should upload this as a separate file labeled 'Revised Manuscript with Track Changes'.
- An unmarked version of your revised paper without tracked changes. You should upload this as a separate file labeled 'Manuscript'.

If applicable, we recommend that you deposit your laboratory protocols in protocols.io to enhance the reproducibility of your results. Protocols.io assigns your protocol its own identifier (DOI) so that it can be cited independently in the future. For instructions see: https://journals.plos.org/plosone/s/submission-guidelines#loc-laboratory-protocols . Additionally, PLOS ONE offers an option for publishing peer-reviewed Lab Protocol articles, which describe protocols hosted on protocols.io. Read more information on sharing protocols at https://plos.org/protocols?utm_medium=editorial-email&utm_source=authorletters&utm_campaign=protocols .

We look forward to receiving your revised manuscript.

Kind regards,

Metodi D Metodiev, Ph.D.

Academic Editor

PLOS ONE

Journal Requirements:

When submitting your revision, we need you to address these additional requirements.

1. Please ensure that your manuscript meets PLOS ONE's style requirements, including those for file naming. The PLOS ONE style templates can be found at

https://journals.plos.org/plosone/s/file?id=wjVg/PLOSOne_formatting_sample_main_body.pdf and

https://journals.plos.org/plosone/s/file?id=ba62/PLOSOne_formatting_sample_title_authors_affiliations.pdf

2. To comply with PLOS ONE submissions requirements, in your Methods section, please provide additional information regarding the experiments involving animals and ensure you have included details on (1) methods of sacrifice, (2) methods of anesthesia and/or analgesia, and (3) efforts to alleviate suffering.

3. We note that the grant information you provided in the ‘Funding Information’ and ‘Financial Disclosure’ sections do not match.

When you resubmit, please ensure that you provide the correct grant numbers for the awards you received for your study in the ‘Funding Information’ section.

4. Thank you for stating the following financial disclosure:

“Academy of medical sciences (SCJ)

NIH GMS (SCJ) (PGM)

NIH NINDS (SCJ)

Northwest Mitochondrial Research Guild (SCJ, AH, BK)”

Please state what role the funders took in the study.  If the funders had no role, please state: "The funders had no role in study design, data collection and analysis, decision to publish, or preparation of the manuscript."

If this statement is not correct you must amend it as needed.

Please include this amended Role of Funder statement in your cover letter; we will change the online submission form on your behalf.

5. Please expand the acronym “NIH” (as indicated in your financial disclosure) so that it states the name of your funders in full.

This information should be included in your cover letter; we will change the online submission form on your behalf.

6. In the online submission form, you indicated that [Insert text from online submission form here].

All PLOS journals now require all data underlying the findings described in their manuscript to be freely available to other researchers, either 1. In a public repository, 2. Within the manuscript itself, or 3. Uploaded as supplementary information.

This policy applies to all data except where public deposition would breach compliance with the protocol approved by your research ethics board. If your data cannot be made publicly available for ethical or legal reasons (e.g., public availability would compromise patient privacy), please explain your reasons on resubmission and your exemption request will be escalated for approval.

7. PLOS requires an ORCID iD for the corresponding author in Editorial Manager on papers submitted after December 6th, 2016. Please ensure that you have an ORCID iD and that it is validated in Editorial Manager. To do this, go to ‘Update my Information’ (in the upper left-hand corner of the main menu), and click on the Fetch/Validate link next to the ORCID field. This will take you to the ORCID site and allow you to create a new iD or authenticate a pre-existing iD in Editorial Manager.

8. Please include your full ethics statement in the ‘Methods’ section of your manuscript file. In your statement, please include the full name of the IRB or ethics committee who approved or waived your study, as well as whether or not you obtained informed written or verbal consent. If consent was waived for your study, please include this information in your statement as well.

9. Please include captions for your Supporting Information files at the end of your manuscript, and update any in-text citations to match accordingly. Please see our Supporting Information guidelines for more information: http://journals.plos.org/plosone/s/supporting-information.

10. Please review your reference list to ensure that it is complete and correct. If you have cited papers that have been retracted, please include the rationale for doing so in the manuscript text, or remove these references and replace them with relevant current references. Any changes to the reference list should be mentioned in the rebuttal letter that accompanies your revised manuscript. If you need to cite a retracted article, indicate the article’s retracted status in the References list and also include a citation and full reference for the retraction notice.

Additional Editor Comments:

The reviewers acknowledge the work’s significance, but require further changes in the form of minor revisions. Please address all points raised by the reviewers.

[Note: HTML markup is below. Please do not edit.]

Reviewers' comments:

Reviewer's Responses to Questions

**Comments to the Author**

1. Is the manuscript technically sound, and do the data support the conclusions?

Reviewer #1: Partly

Reviewer #2: No

Reviewer #3: Yes

**********

2. Has the statistical analysis been performed appropriately and rigorously?

Reviewer #1: Yes

Reviewer #2: N/A

Reviewer #3: I Don't Know

**********

3. Have the authors made all data underlying the findings in their manuscript fully available?

The PLOS Data policy

Reviewer #1: No

Reviewer #2: Yes

Reviewer #3: Yes

**********

4. Is the manuscript presented in an intelligible fashion and written in standard English?

Reviewer #1: Yes

Reviewer #2: Yes

Reviewer #3: Yes

**********

Reviewer #1: Hanaford and colleagues studied the role of lymphocytes in the development of the phenotype of Ndufs4(-/-) mice. Ndufs4 KO is a mouse model of Leigh syndrome characterized by subacute necrotizing encephalomyopathy, recapitulated in mice. Recent work, namely from the same group, has determined that leukocytes, in particular peripheral macrophages, rather than microglia were involved in pathogenesis (PMID: 37552802). Furthermore, IFN gamma was also implicated (PMID: 38680020), whereas the role of IL-6 was limited (PMID: 35770802).

To interrogate the role of the adaptive immune system and lymphocytes, which could be the source of IFN gamma, the authors crossed the Ndufs4 KO mouse to the B, T and NK cell-depleted mouse model, due to deletion of X chromosome encoded Il2rg. No effect on weight loss, ataxia or survival could be detected, suggesting that lymphocytes are not involved in neurological pathogenesis.

These new results add to the previous body of work of the group and others dissecting the immune cells involved in the Ndufs4 KO phenotype, using relevant animal groups, which will be useful to the community. Further details on the immunodeficient animals, discussion of published results and the implications of the findings would improve this manuscript.

-Although the Il2rg model has already been published, some aspects of the verification of lymphocyte depletion are missing or confusing. The count of NK in Supp1 is unchanged whereas the % is in Fig 1. How do the authors explain this? How does CD45 gating look in Supp1? Is the total CD45+ cell count decreased? Is there compensatory increase or activation in the myeloid compartment?

Importantly, are the findings similar in the context of Ndufs4 KO?

-Similarly, is the phenotype of macrophages changed due to the absence of lymphocytes, which could confound results?

-The source of IFNg, which has been implicated in Ndufs4 KO mouse phenotype, is mainly T cells and NK cells. How do the authors explain this apparent contradiction?

-The authors discuss the evidence for immunodeficiency in mitochondrial disease, in particular for Leigh syndrome. However, more relevant to their findings of the immune system contributing to disease, more and more reports suggest that the innate immune system may be activated in several mitochondrial disease, in particular type I interferon signalling (e.g. PMID: 36635485, PMID: 34387651). This could be consistent with the lack of a role for IL-6. This aspect deserves a mention in the discussion.

Minor:

-Concerning the survival in Figure 3, Il2rg +/- animals seems to have a longer survival, which may be significant. However, they are only females. The table should show males and females separately for a more rigorous comparison.

-The nomenclature of Il2rg KO animals varies in the manuscript: -/- in methods, KO in figures and X/Y in Figure 3. This should be homogenized.

-Line 96, is the genotype of the male breeder right: Ndufs4(-/-)/Il2rg(-/Y)?

-FACS antibodies are missing from methods

-Line 125: Il2rg genotype is missing in the paragraph

-Line 152: B200 written instead of B220

-Supp2 legend has mistakes.

-Fig2E is not referenced in the text.

-Fig3: what does FDIC mean?

-There are some typos along the text.

Reviewer #2: This short manuscript reported a negative finding that disruption of adaptive immunity by IL2Rg KO did not attenuate Leigh Syndrome mice with Ndufs4 KO. Reports of negative findings can be very important if there is a widespread and unproven belief. Unfortunately, I do not find a strong rationale for the proposed experiments. In other words, there is little evidence or clues that Leigh Syndrome will involve adaptive immunity. Adaptive immunity is very critical to protect against infection, spreading of cancer and its overactivity is associated with autoimmune diseases. Leigh Syndrome is a genetic disease involving mitochondria, presented as neurodegenerative, early in life. While a few manuscripts have been published to report the involvement of microglia (including the report by the current authors), the reasons to do such experiments are not strongly supported by previous literature. As such, the value of this short manuscript reporting negative findings is very limited. Most importantly, CNS has an immune privilege due to the blood brain barrier. It is unclear why they did a systemic KO of IL2Rg as there is no strong evidence of systemic inflammation in Leigh Syndrome. A more thoughtful experiments addressing mechanisms of neuroinflammation may be much more exciting.

Reviewer #3: This is a well thought out and well written manuscript on a worthwhile topic.

I have a few comments and suggestions:

-Could the authors please provide the figure legends with the statistic test details? I cannot find them in the pdf provided. I would also favor including these details in the methods section.

-Regarding the exclusion criteria. In light of the data presenting regarding weight loss in the analyzed animals, could the authors please provide details by genotype regarding how many were excluded due to eutanasia prior to age of Ndufs4 disease onset and how many by genotype were excluded in early life due to being runts? It seems possible that the double KO may have worsened this aspect of the presentation in mice. While I recognize that the authors are choosing to focus on the absence of clear amelioration, this data would be of interest.

-Additional discussion of the imrpoved survival in the Ndufs4 KO/Il2rg het animals would be appreciated.

Minor points:

-Please add "and/or the basal ganglia" to the definition presented in the 2nd sentence of the abstract per citation #8 (McCormick et al).

-The authors might want to consider adding 2 citations to their discussion of the adaptive immune system perturbations in human GMD patients: PMID 37104980 (limitations in B cell repertoire in GMD patients) and PMID 38499449 (T cell repertoire oligoclonality in MELAS patients).

**********

what does this mean? ). If published, this will include your full peer review and any attached files.

If you choose “no”, your identity will remain anonymous but your review may still be made public.

**Do you want your identity to be public for this peer review?** For information about this choice, including consent withdrawal, please see our Privacy Policy

Reviewer #1: No

Reviewer #2: No

Reviewer #3: No

**********

[NOTE: If reviewer comments were submitted as an attachment file, they will be attached to this email and accessible via the submission site. Please log into your account, locate the manuscript record, and check for the action link "View Attachments". If this link does not appear, there are no attachment files.]

While revising your submission, please upload your figure files to the Preflight Analysis and Conversion Engine (PACE) digital diagnostic tool, https://pacev2.apexcovantage.com/ . PACE helps ensure that figures meet PLOS requirements. To use PACE, you must first register as a user. Registration is free. Then, login and navigate to the UPLOAD tab, where you will find detailed instructions on how to use the tool. If you encounter any issues or have any questions when using PACE, please email PLOS at figures@plos.org

Attachment Submitted filename: LS adaptive immunity.docx

# Author comment: Author response to Decision Letter 1

**DOI:** 10.1371/journal.pone.0324268.r003

19 Feb 2025

Manuscript PONE-D-24-54394

Responses to reviewer comments

Overview:

We thank the reviewers for their thoughtful remarks and recommendations. The comments of two of the three reviewers were very positive and support our view that these findings will be of interest to the readers of Orphanet Journal of Rare Diseases. Our responses are given below, and we have submitted a revised version of the manuscript.

We addressed each of the comments point-by-point as detailed below, in order of shortest to longest review:

Reviewer 1

Hanaford and colleagues studied the role of lymphocytes in the development of the phenotype of Ndufs4(-/-) mice. Ndufs4 KO is a mouse model of Leigh syndrome characterized by subacute necrotizing encephalomyopathy, recapitulated in mice. Recent work, namely from the same group, has determined that leukocytes, in particular peripheral macrophages, rather than microglia were involved in pathogenesis (PMID: 37552802). Furthermore, IFN gamma was also implicated (PMID: 38680020), whereas the role of IL-6 was limited (PMID: 35770802).

We thank the reviewer for their positive and thoughtful notes. While not an actionable point, we clarify that we have demonstrated peripheral macrophages are involved in disease and capable of driving pathology in the absence of microglia, not that microglia are not involved.

To interrogate the role of the adaptive immune system and lymphocytes, which could be the source of IFN gamma, the authors crossed the Ndufs4 KO mouse to the B, T and NK cell-depleted mouse model, due to deletion of X chromosome encoded Il2rg. No effect on weight loss, ataxia or survival could be detected, suggesting that lymphocytes are not involved in neurological pathogenesis.

These new results add to the previous body of work of the group and others dissecting the immune cells involved in the Ndufs4 KO phenotype, using relevant animal groups, which will be useful to the community. Further details on the immunodeficient animals, discussion of published results and the implications of the findings would improve this manuscript.

Although the Il2rg model has already been published, some aspects of the verification of lymphocyte depletion are missing or confusing. The count of NK in Supp1 is unchanged whereas the % is in Fig 1. How do the authors explain this? How does CD45 gating look in Supp1? Is the total CD45+ cell count decreased? Is there compensatory increase or activation in the myeloid compartment?

We apologize for any lack of clarity in the cytometry data. To address this, we have added a supplemental figure showing the gating approach, for B-, T-, and NK- cells we have now provided both total cell counts per mL of blood (in Figure 1) and counts normalized to CD45 cell numbers (in Supplemental Figure 1). These data show that CD45 cell numbers are indeed decreased overall (see flow gating charts).

Importantly, are the findings similar in the context of Ndufs4 KO? Similarly, is the phenotype of macrophages changed due to the absence of lymphocytes, which could confound results? The source of IFNg, which has been implicated in Ndufs4 KO mouse phenotype, is mainly T cells and NK cells. How do the authors explain this apparent contradiction?

We agree that further exploration of the impact of B-, T-, and NK- cell depletion on macrophages might be of interest in other settings, but as we have answered our primary query in regard to Leigh syndrome, this is beyond the scope of this study. If such changes occur, they would not be relevant to disease in patients, but perhaps could account for our observations that the Ndufs4(-/-)/Il2rg(KO) mice appear slightly worse off than Ndufs4(-/-)/Il2rg(+/-) animals. However, a change in macrophage activation would not confound our key findings, which are that the major adaptive immune players are not responsible for disease (i.e. disease progresses without these cellular components of the adaptive immune system, and activation of macrophages is alone not sufficient to cause LS). We agree that the point suggesting macrophage activation explains the slightly shortened Il2rg(KO); to address this comment we’ve added the following paragraph to the discussion “In the cohorts tested here, we observed a modest but statistically significant reduction in Ndufs4(-/-)/Il2rg(KO) compared to Ndufs4(-/-)/Il2rg(+/-) animals. This might indicate that the Ndufs4(-/-)/Il2rg(KO) mice are more frail than Ndufs4(-/-)/Il2rg(+/-) animals, which would be consistent with the possibility that adaptive immune depletion not only fails to prevent disease but is actively harmful to the Leigh syndrome model. It is also possible that loss of Il2rg impacts the phenotype of macrophages, possibly slightly accelerating disease through increased activation. This may be worth further study.”

Regarding the source of IFNg: this is an interesting point worth discussing. To address this comment, we have added the following text to the discussion: “In recent work, we found that loss of IFN� provided a modest but statistically significant and gene dose-dependent improvement in survival and delay in symptom onset in Ndufs4(-/-) mice (13). Notably, cytotoxic lymphocytes, including T- and NK- cells, and cytotoxic macrophages are the major producers of IFN� (20, 21). Given that IFN� depletion attenuates disease progression, but B-, T-, and NK- cell depletion fails to alter disease course, it seems likely that disease-promoting IFN� is macrophage-derived, representing positive feedback.”

The authors discuss the evidence for immunodeficiency in mitochondrial disease, in particular for Leigh syndrome. However, more relevant to their findings of the immune system contributing to disease, more and more reports suggest that the innate immune system may be activated in several mitochondrial disease, in particular type I interferon signalling (e.g. PMID: 36635485, PMID: 34387651). This could be consistent with the lack of a role for IL-6. This aspect deserves a mention in the discussion.

We thank the reviewer for this thoughtful comment; we reviewed this topic recently, and agree it is important and relevant. Regarding IL-6, this factor doesn’t seem to be a primary driver of disease, but the Quintana laboratory published some data suggesting it contributes to overall disease progression – perhaps similar to our findings with IFN gamma, it is one of many disease driving cytokines. We’ve added the following text to the discussion to address this point, at the beginning of the paragraph referenced: “Recent evidence supports the notion that activation of the innate immune system may occur in multiple forms of mitochondrial disease (4, 22, 23). Various contributing pathways have been implicated, such as type I interferon signaling, but no clear single immune-activating mediator has yet been identified.”

Minor:

-Concerning the survival in Figure 3, Il2rg +/- animals seems to have a longer survival, which may be significant. However, they are only females. The table should show males and females separately for a more rigorous comparison.

We thank the reviewer for noting this point of interest. To address the reviewer’s comment, we have added a panel to Figure 3 showing the comparison between Ndufs4(-/-) mice split by sex and statistical analysis to panel D. This comparison was not statistically significant, but it we agree it is worthwhile to include this. We have also added to this figure a reminder to readers that the Il2rg(+/-) mice are all female (as it is X-linked).

-The nomenclature of Il2rg KO animals varies in the manuscript: -/- in methods, KO in figures and X/Y in Figure 3. This should be homogenized.

We have carefully proof-read the manuscript to ensure consistency and have added addiitonal text to the method to more clearly state this. In the methods we use X/Y nomenclature is used to designate breeder genotype with X/Y status specifically noted and define the use of KO vs +/-. In the remainder of the manuscript uses Ndufs4(+/-) and Il2rg(KO) to designate female hets (only females can be het, given the gene is X-linked) and KO (both male -/Y and -/- female) mice.

-Line 96, is the genotype of the male breeder right: Ndufs4(-/-)/Il2rg(-/Y)?

Yes – knockout males carry one Il2rg null allele X-chromosome and one Y chromosome. We have clarified the nomenclature in the methods by adding X and Y to the chromosomes of the parent breeders.

-FACS antibodies are missing from methods

-Line 125: Il2rg genotype is missing in the paragraph

-Line 152: B200 written instead of B220

-Supp2 legend has mistakes.

-Fig2E is not referenced in the text.

-Fig3: what does FDIC mean?

-There are some typos along the text.

We have addressed these errors and thank the reviewer for their careful notes.

Reviewer 2

This short manuscript reported a negative finding that disruption of adaptive immunity by IL2Rg KO did not attenuate Leigh Syndrome mice with Ndufs4 KO. Reports of negative findings can be very important if there is a widespread and unproven belief. Unfortunately, I do not find a strong rationale for the proposed experiments. In other words, there is little evidence or clues that Leigh Syndrome will involve adaptive immunity. Adaptive immunity is very critical to protect against infection, spreading of cancer and its overactivity is associated with autoimmune diseases. Leigh Syndrome is a genetic disease involving mitochondria, presented as neurodegenerative, early in life.

As the other reviewers have noted, this work, while negative, answers an important question in a line of work our lab has been undertaking. This is detailed at length in the introduction and discussion and we will not repeat the text here, but will note key points:

-Our lab demonstrated recently that depletion of CSF1R positive immune cells prevents Leigh syndrome in the Ndufs4(KO) model. This finding provided an extremely important breakthrough in our understanding of LS, but the precise sub-population (or populations) of immune cells driving disease has/have not yet been established.

-Our lab has recently shown that Leigh syndrome is driven by peripheral immune cells, and as such the lesions in Leigh syndrome are not ‘immune privileged.’

“While a few manuscripts have been published to report the involvement of microglia (including the report by the current authors), the reasons to do such experiments are not strongly supported by previous literature. As such, the value of this short manuscript reporting negative findings is very limited. Most importantly, CNS has an immune privilege due to the blood brain barrier. It is unclear why they did a systemic KO of IL2Rg as there is no strong evidence of systemic inflammation in Leigh Syndrome. A more thoughtful experiments addressing mechanisms of neuroinflammation may be much more exciting.”

We are particularly mystified by these comments. Regardless of the reviewer’s views on our prior rationale for the 2022 study, the findings are hard to disregard – we demonstrated that depletion of immune cells fully suppresses Leigh syndrome disease pathology in the Ndufs4(-/-) mouse model. This has led to follow-up work showing that peripheral immune cells drive disease in the absence of microglia (Hanaford et al, 2023). Our work has very clearly shown Leigh syndrome is immune mediated. The importance of these findings is recognized by the mitochondrial disease research community and the rationale for continued work in this area is very robust.

We believe this reviewer did not read the introduction or discussion (perhaps also skipping the abstract) and is not up to date on the field, as the answers to these criticisms are provided at length. Reviewers 1 and 3 strongly support the rationale for this study and support publication of our findings. Given that reviewer 2 provided no critiques apart from not understanding the background in the field, we have not modified our manuscript in response to the comments from reviewer 2.

Reviewer 3

This is a well thought out and well written manuscript on a worthwhile topic.

We thank the reviewer for their positive comments.

I have a few comments and suggestions:

-Could the authors please provide the figure legends with the statistic test details? I cannot find them in the pdf provided. I would also favor including these details in the methods section.

We apologize for any omissions; in the revision, we have ensured that each figure legend states the tests clearly, and have added additional details to the method section under ‘statistical analyses’.

-Regarding the exclusion criteria. In light of the data presenting regarding weight loss in the analyzed animals, could the authors please provide details by genotype regarding how many were excluded due to eutanasia prior to age of Ndufs4 disease onset and how many by genotype were excluded in early life due to being runts? It seems possible that the double KO may have worsened this aspect of the presentation in mice. While I recognize that the authors are choosing to focus on the absence of clear amelioration, this data would be of interest.

We apologize for any lack of clarity, and agree this point deserves expansion. The inclusion criteria are provided to ensure that any labs reproducing our studies will obtain the same results, but these criteria are fairly standard for mouse studies and we did not observe any differences in rates of exclusion by genotype. We have expanded the exclusion criteria as follows:

“Only animals with a PCR confirmed genotype were included in the study. Animals euthanized prior to the age of disease onset in the Ndufs4(-/-) were excluded from study. Our criteria for early life euthanasia and study exclusion include severe weaning stress (significant weight loss or death prior to P30), runts (defined as ≤5 g body weight at weaning age), or health issues unrelated to the Ndufs4(-/-) phenotype (such as hydrocephalus). These criteria are applied to all genotypes as part of our standard animal care. Exclusion due to these criteria occurs in up to ~10% of Ndufs4(-/-) animals in our prior studies. From Ndufs4(+/-)/Il2rg(+/-) x Ndufs4(-/-)/Il2rg(-/Y) and Ndufs4(+/-)/Il2rg(+/-) x Ndufs4(+/-)/Il2rg(+/Y) matings, 1 of 10 Ndufs4(-/-)/Il2rg(KO) and 1 of 8 Ndufs4(-/-)/Il2rg(WT) were excluded, similar to our overall exclusion rate for Ndufs4(-/-) mice.”

We cannot rule out the possibility that a small difference in runt rate, etc, might be observed if very large numbers of animals were used, but did not observe any differences in the cohorts in this study.

-Additional discussion of the improved survival in the Ndufs4 KO/Il2rg het animals would be appreciated.

We thank the reviewer for this comment; while we don’t wish to overinterpret this observation, we agree it is worth noting in the discussion. We have added the following text:

“In the cohorts tested here, we observed a modest but statistically significant reduction in Ndufs4(-/-)/Il2rg(KO) compared to Ndufs4(-/-)/Il2rg(+/-) animals. This might indicate that the Ndufs4(-/-)/Il2rg(KO) mice are more frail than Ndufs4(-/-)/Il2rg(+/-) animals, which would be consistent with the possibility that adaptive immune depletion not only fails to prevent disease but is actively harmful to the Leigh syndrome model. This may be worth further study.”

Minor points:

-Please add "and/or the basal ganglia" to the definition presented in the 2nd sentence of the abstract per citation #8 (McCormick et al).

-The authors might want to consider adding 2 citations to their discussion of the adaptive immune system perturbations in human GMD patients: PMID 37104980 (limitations in B cell repertoire in GMD patients) and PMID 38499449 (T cell repertoire oligoclonality in MELAS patients).

We have added this to the definition and have added these citations.

# Peer review: Decision Letter 1

**DOI:** 10.1371/journal.pone.0324268.r004

**Authors:** Metodi Metodiev

9 Apr 2025

Dear Dr. Johnson,

Please submit your revised manuscript by May 24 2025 11:59PM. If you will need more time than this to complete your revisions, please reply to this message or contact the journal office at plosone@plos.org . When you're ready to submit your revision, log on to https://www.editorialmanager.com/pone/ and select the 'Submissions Needing Revision' folder to locate your manuscript file.

- A rebuttal letter that responds to each point raised by the academic editor and reviewer(s). You should upload this letter as a separate file labeled 'Response to Reviewers'.
- A marked-up copy of your manuscript that highlights changes made to the original version. You should upload this as a separate file labeled 'Revised Manuscript with Track Changes'.
- An unmarked version of your revised paper without tracked changes. You should upload this as a separate file labeled 'Manuscript'.

If applicable, we recommend that you deposit your laboratory protocols in protocols.io to enhance the reproducibility of your results. Protocols.io assigns your protocol its own identifier (DOI) so that it can be cited independently in the future. For instructions see: https://journals.plos.org/plosone/s/submission-guidelines#loc-laboratory-protocols . Additionally, PLOS ONE offers an option for publishing peer-reviewed Lab Protocol articles, which describe protocols hosted on protocols.io. Read more information on sharing protocols at https://plos.org/protocols?utm_medium=editorial-email&utm_source=authorletters&utm_campaign=protocols .

We look forward to receiving your revised manuscript.

Kind regards,

Metodi D Metodiev, Ph.D.

Academic Editor

PLOS ONE

Journal Requirements:

Please review your reference list to ensure that it is complete and correct. If you have cited papers that have been retracted, please include the rationale for doing so in the manuscript text, or remove these references and replace them with relevant current references. Any changes to the reference list should be mentioned in the rebuttal letter that accompanies your revised manuscript. If you need to cite a retracted article, indicate the article’s retracted status in the References list and also include a citation and full reference for the retraction notice.

Additional Editor Comments:

Both reviewers have recommended the acceptance of the article. However, before proceeding with formal acceptance, I request the authors to address the concerns raised by Reviewer 1.

[Note: HTML markup is below. Please do not edit.]

Reviewers' comments:

Reviewer's Responses to Questions

**Comments to the Author**

Reviewer #1: (No Response)

Reviewer #3: All comments have been addressed

**********

2. Is the manuscript technically sound, and do the data support the conclusions?

Reviewer #1: Yes

Reviewer #3: Yes

**********

3. Has the statistical analysis been performed appropriately and rigorously?

Reviewer #1: Yes

Reviewer #3: Yes

**********

4. Have the authors made all data underlying the findings in their manuscript fully available?

The PLOS Data policy

Reviewer #1: Yes

Reviewer #3: (No Response)

**********

5. Is the manuscript presented in an intelligible fashion and written in standard English?

Reviewer #1: Yes

Reviewer #3: Yes

**********

Reviewer #1: The authors have satisfactorily addressed my main comments, which I thank them for.

I would just point out:

- Although mention of Y is necessary, I am not sure mention of “X” in genotypes is necessary, now that the genotypes are well clarified.

- FACS antibodies should mention the clone and fluorochrome used.

- New version line 88: I think it should be “homozygous mutant female (IL2rg(X-/X-))” rather than (X+/X-)

I also don’t understand this statement in the rebuttal, but I think KO should read +/- for females:

“In the remainder of the manuscript uses Ndufs4(+/-) and Il2rg(KO) to designate female hets (only females can be het, given the gene is X-linked) and KO (both male -/Y and -/- female) mice.”

- New version line 154: I think the genotype of the male breeder should be Ndufs4(+/-) Il2rg (-/Y) and not Ndufs4(-/-) Il2rg (-/Y), as Ndufs4(-/-) animals can apparently not breed.

- Line 207 should refer to Fig 3D I think.

- New version line 242: the main word is missing (“survival”?).

- I think figure 3B and Supp 3 are the same (and fig supp 3 not referred to by the text).

Reviewer #3: The authors' responses to my comments and those of other reviewers is satisfactory. I particularly appreciate the depth added to the discussion by their responses to the comments from Reviwer#1. I stronly support, in general, the reporting of negative findings in robust studies as this is lacking in science broadly and holds back progress generally.

**********

what does this mean? ). If published, this will include your full peer review and any attached files.

If you choose “no”, your identity will remain anonymous but your review may still be made public.

**Do you want your identity to be public for this peer review?** For information about this choice, including consent withdrawal, please see our Privacy Policy

Reviewer #1: No

Reviewer #3: No

**********

[NOTE: If reviewer comments were submitted as an attachment file, they will be attached to this email and accessible via the submission site. Please log into your account, locate the manuscript record, and check for the action link "View Attachments". If this link does not appear, there are no attachment files.]

While revising your submission, please upload your figure files to the Preflight Analysis and Conversion Engine (PACE) digital diagnostic tool, https://pacev2.apexcovantage.com/ . PACE helps ensure that figures meet PLOS requirements. To use PACE, you must first register as a user. Registration is free. Then, login and navigate to the UPLOAD tab, where you will find detailed instructions on how to use the tool. If you encounter any issues or have any questions when using PACE, please email PLOS at figures@plos.org

# Author comment: Author response to Decision Letter 2

**DOI:** 10.1371/journal.pone.0324268.r005

14 Apr 2025

Manuscript PONE-D-24-54394

Responses to reviewer comments

Overview:

We thank the reviewers for their thoughtful remarks. We have addressed the final comments from Reviewer 1 as follows, detailed point by point:

Reviewer 1

Reviewer #1: The authors have satisfactorily addressed my main comments, which I thank them for.

I would just point out:

Although mention of Y is necessary, I am not sure mention of “X” in genotypes is necessary, now that the genotypes are well clarified.

We thank the reviewer for their positive and thoughtful notes and careful review. Their feedback has been extremely productive throughout the review process.

As this point is optional, we have opted to keep X in the genotypes, as we feel it is helpful in keeping track of the genotypes and sexes.

FACS antibodies should mention the clone and fluorochrome used.

We apologize for this omission; we have added these details and the dilutions to the methods section as a supplemental table. They are as follows:

Name Fluor Clone Dilution

anti-CD45 AF488 30-F11 1:100

anti-CD3 APC 145-2C11 1:100

anti-B220 PE RA3-6B2 1:100

anti-NK1.1 PE-Cy7 PK136 1:100

We have also added the dilution and catalog number for the dead cell Zombie dye from BioLegend (cat. #423101, used at a 1:200 dilution).

New version line 88: I think it should be “homozygous mutant female (IL2rg(X-/X-))” rather than (X+/X-)

We thank the reviewer for catching this typo, it has been fixed.

I also don’t understand this statement in the rebuttal, but I think KO should read +/- for females:

“In the remainder of the manuscript uses Ndufs4(+/-) and Il2rg(KO) to designate female hets (only females can be het, given the gene is X-linked) and KO (both male -/Y and -/- female) mice.”

We apologize for the lack of clarity in our response statement; the relevant sentence in the text correctly states “Il2rg is encoded on the X chromosome, so Il2rg(KO) indicates homozygous knockout female (IL2rg(X+/X-)) or hemizygous knockout male (IL2rg(X-/Y)).” This was noted in the methods section and we have added it to the beginning of the results in this final revision. While there was a typo in our response, as the reviewer notes above the genotypes are well clarified in the last revision.

New version line 154: I think the genotype of the male breeder should be Ndufs4(+/-) Il2rg (-/Y) and not Ndufs4(-/-) Il2rg (-/Y), as Ndufs4(-/-) animals can apparently not breed.

We thank the reviewer for noting this typographical error; they are correct, and we have fixed the text.

Line 207 should refer to Fig 3D I think.

We thank the reviewer for noting this typographical error; they are correct, and we have fixed the text.

New version line 242: the main word is missing (“survival”?)

We thank the reviewer for noting this typographical error; they are correct, and we have fixed the text.

I think figure 3B and Supp 3 are the same (and fig supp 3 not referred to by the text)

This is correct, we appear to have accidentally left a duplicate of this panel in the manuscript file as supplemental, after it was included in the main figure. This duplicate has now been removed.

Attachment Submitted filename: Responses to reviewer comments.docx

# Editor report: Decision Letter 2

**DOI:** 10.1371/journal.pone.0324268.r006

**Authors:** Metodi Metodiev

23 Apr 2025

Disruption of adaptive immunity does not attenuate disease in the Ndufs4(-/-) model of Leigh syndrome

PONE-D-24-54394R2

Dear Dr. Johnson,

We’re pleased to inform you that your manuscript has been judged scientifically suitable for publication and will be formally accepted for publication once it meets all outstanding technical requirements.

Within one week, you’ll receive an e-mail detailing the required amendments. When these have been addressed, you’ll receive a formal acceptance letter and your manuscript will be scheduled for publication.

An invoice will be generated when your article is formally accepted. Please note, if your institution has a publishing partnership with PLOS and your article meets the relevant criteria, all or part of your publication costs will be covered. Please make sure your user information is up-to-date by logging into Editorial Manager at Editorial Manager®  and clicking the ‘Update My Information' link at the top of the page. If you have any questions relating to publication charges, please contact our Author Billing department directly at authorbilling@plos.org.

If your institution or institutions have a press office, please notify them about your upcoming paper to help maximize its impact. If they’ll be preparing press materials, please inform our press team as soon as possible -- no later than 48 hours after receiving the formal acceptance. Your manuscript will remain under strict press embargo until 2 pm Eastern Time on the date of publication. For more information, please contact onepress@plos.org.

Kind regards,

Metodi D Metodiev, Ph.D.

Academic Editor

PLOS ONE

Additional Editor Comments (optional):

The authors have adequately addressed all comments from the reviewer. I thank them for their responsiveness and perseverance.

Reviewers' comments:

# Editor report: Acceptance letter

**DOI:** 10.1371/journal.pone.0324268.r007

**Authors:** Metodi Metodiev

PONE-D-24-54394R2

PLOS ONE

Dear Dr. Johnson,

I'm pleased to inform you that your manuscript has been deemed suitable for publication in PLOS ONE. Congratulations! Your manuscript is now being handed over to our production team.

At this stage, our production department will prepare your paper for publication. This includes ensuring the following:

* All references, tables, and figures are properly cited

* All relevant supporting information is included in the manuscript submission,

* There are no issues that prevent the paper from being properly typeset

You will receive further instructions from the production team, including instructions on how to review your proof when it is ready. Please keep in mind that we are working through a large volume of accepted articles, so please give us a few days to review your paper and let you know the next and final steps.

Lastly, if your institution or institutions have a press office, please let them know about your upcoming paper now to help maximize its impact. If they'll be preparing press materials, please inform our press team within the next 48 hours. Your manuscript will remain under strict press embargo until 2 pm Eastern Time on the date of publication. For more information, please contact onepress@plos.org.

If we can help with anything else, please email us at customercare@plos.org.

Thank you for submitting your work to PLOS ONE and supporting open access.

Kind regards,

PLOS ONE Editorial Office Staff

on behalf of

Dr. Metodi D Metodiev

Academic Editor

PLOS ONE
